# A Case Report of Non‐Neutralizing Acquired Factor V Inhibitor Mimicking Deficiency: Diagnostic Challenges and Therapeutic Implications

**DOI:** 10.1155/crh/4306324

**Published:** 2026-01-08

**Authors:** Shreyas Kalantri, Pranali Pachika, Shiva Balasubramanian, Bayan Alquran, Morgan McCoy, Vivek Sharma

**Affiliations:** ^1^ Division of Hematology and Oncology, Brown Cancer Center, University of Louisville, Louisville, Kentucky, USA, louisville.edu; ^2^ Department of Medicine, University of Louisville, Louisville, Kentucky, USA, louisville.edu; ^3^ Department of Pathology and Laboratory Medicine, University of Louisville, Louisville, Kentucky, USA, louisville.edu

**Keywords:** acquired Factor V deficiency, coagulopathy, Factor V inhibitor, hematuria, immunosuppressive therapy, intravenous immunoglobulin (IVIG), non-neutralizing antibody, platelet transfusion, rare bleeding disorder, rituximab

## Abstract

Acquired Factor V (FV) deficiency due to inhibitors is a rare coagulopathy that presents significant diagnostic and therapeutic challenges. We report the case of an 81‐year‐old male with persistent gross hematuria and severe coagulopathy, marked by prolonged prothrombin time (PT), activated partial thromboplastin time (aPTT), and critically low FV activity (< 1%). Initial mixing studies corrected the coagulation abnormalities, suggesting a deficiency rather than an inhibitor; however, standard therapies failed. Fresh frozen plasma (FFP) did not elevate FV levels, and recombinant activated Factor VII (rFVIIa) did not resolve his symptoms, raising suspicion for a non‐neutralizing inhibitor that depletes FV by increasing clearance. Clinical improvement was achieved with platelet transfusions, and his FV level normalized after treatment with rituximab and intravenous immunoglobulin (IVIG). PT and aPTT improved from 60 and > 200 to 12 and 32, respectively. It has remained normal with subsequent maintenance immunosuppression with rituximab. This case illustrates the diagnostic complexity created by non‐neutralizing FV inhibitors, which accelerate factor clearance without directly impairing activity. It highlights the critical need for integrating clinical and laboratory findings to guide tailored treatment in managing rare coagulopathies.

## 1. Introduction

Acquired Factor V (FV) deficiency due to inhibitors is a rare condition, with an estimated incidence of 0.09–0.29 cases per million person‐years [1]. Diagnosing and treating FV deficiency is challenging due to variable bleeding severity and inconsistent lab–clinical correlation. Unlike inherited cases, acquired FV deficiency results from auto‐ or alloantibodies that inhibit or accelerate FV clearance [1, 2].

This case is notable due to the presence of a suspected non‐neutralizing FV inhibitor, which led to severe coagulopathy unresponsive to standard treatments. Initial mixing studies, due to the non‐neutralizing nature of the inhibitor, indicated a simple factor deficiency, which delayed the recognition of the inhibitor. Once the diagnosis was suspected, based on the lack of response to plasma‐based therapies and bypassing agents, a stepwise treatment approach was made, involving platelet transfusions followed by immunosuppression with rituximab and IVIG. This approach ultimately resolved the symptoms and normalized the coagulation parameters. This case report offers valuable insights into the diagnostic challenges and treatment strategies for managing patients with acquired FV inhibitors, especially when non‐neutralizing mechanisms are suspected.

## 2. Case Summary

An 81‐year‐old male with a history of hypertension and Stage I follicular lymphoma (treated with excision and a watch‐and‐wait approach) presented with worsening gross hematuria and severe coagulopathy over three weeks. He was transferred to our hospital after experiencing a fall at home, with initial laboratory findings, as detailed in Table 1, revealing severe anemia (hemoglobin: 6.3 g/dL) and markedly prolonged coagulation parameters, including an INR of 5.46. CT urography did not show wall thickening or masses in the bladder. Despite receiving 105 mg total of Vitamin K through PO and IV routes and 2U of fresh frozen plasma (FFP) at the referring facility, his hematuria persisted.

**Table 1:** Initial laboratory evaluation and mixing study.

Test	Patient values (reference range)
Hemoglobin (g/dL)	6.3 (13–17.5)
Platelet (× 10(3)/μL)	249 (140–420)
Prothrombin time (sec.)	60 (9.0–11.5)
INR	5.46
aPTT (sec.)	> 200 (≤ 40)
1:1 Mixing Study Results	
Prothrombin time	9.6 sec. (≤ 11.5 sec.)
aPTT‐LA (sec.), immediate mix	33 sec. (cutoff 35 sec.)
aPTT‐LA (sec.), incubated mix	37 sec. (cutoff 37 sec.)
Fibrinogen (mg/dL)	386 (230–450)
Von Willebrand factor (%)	127 (50–217)
Ristocetin cofactor (%)	81 (42–200)
Factor assay	
Factor II (%)	75 (70–120)
Factor V (%)	< 1 (65–150)
Factor VII (%)^∗^	51 (65–140)
Factor VIII (%)	134 (70–150)
Factor IX (%)	100 (70–120)
Factor X (%)	81 (70–150)
Factor XI (%)	85 (65–150)
Factor XII (%)	61 (50–150)
Prekallikrein (%)	66 (55–207)
HMWK (%)	76 (65–135)
Serum protein electrophoresis (SPEP)	Normal

^∗^Measured once, not serially repeated.

On admission, physical examination was unremarkable aside from gross hematuria. Laboratory tests confirmed severe coagulopathy, with PT > 60 s, PTT > 100 s, and critically low FV activity (< 1%) which resulted on Day 5. Platelet count, fibrinogen, and D‐dimer were normal, ruling out disseminated intravascular coagulation. Mixing studies initially showed correction of PT and PTT, suggesting a factor deficiency rather than an inhibitor. Serum protein electrophoresis and immunofixation electrophoresis were normal, ruling out monoclonal gammopathy or paraproteinemia as contributing factors. There was no evidence of lymphadenopathy on his imaging.

**Figure 1:**
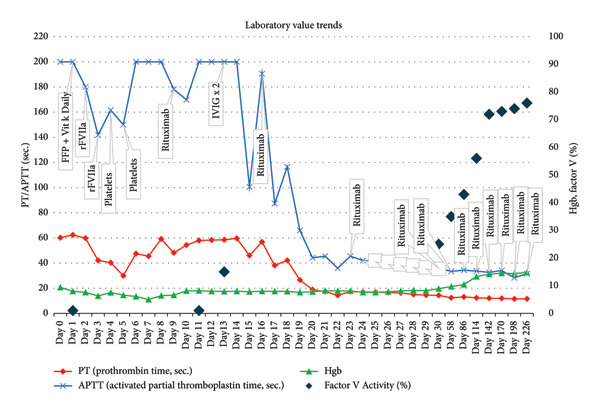
Trends in PT, aPTT, Hgb, and Factor V activity in relation to FFP, rFVIIa, pRBC transfusions, platelet transfusions, rituximab, and IVIG administration over time.

Initial management focused on Vitamin K deficiency and volume depletion, with FFP and bladder irrigation, as the cause of coagulopathy was unknown. Despite these measures, hematuria continued, and coagulation studies remained abnormal. Recombinant activated Factor VII (rFVIIa) was given every six hours to bypass the suspected coagulopathy at a dosage of 75 mcg/kg for 5 days, but hematuria persisted. FV activity, drawn on Day 1 prior to FFP administration, later resulted on Day 5 and was found to be < 1%. At that time, two platelet transfusions were administered after withholding rFVIIa to assess their effect, as platelets are a source of FV. This led to a noticeable improvement in hematuria, indicating that platelet transfusion helped replenish depleted FV levels. Trends in PT, PTT, INR, and FV activity during these interventions are detailed in Figure 1.

Three mixing studies with 2‐h incubation times were obtained during the patient’s hospitalization: two prior to the initiation of rFVIIa and one approximately 24 h after the final rFVIIa dose. All demonstrated correction of PT and aPTT, which initially suggested a factor deficiency and argued against the presence of a neutralizing inhibitor. However, the patient’s persistent bleeding, extremely low FV activity (< 1%), and lack of response to FFP raised concern for an underlying inhibitor. FV activity was confirmed to be < 1% and a simultaneously measured Bethesda assay showed no detectable neutralizing activity (titer < 0.4 BU). Although negative, the Bethesda assay is not validated for FV and may fail to detect non‐neutralizing antibodies that act via accelerated clearance [3]. The discordance between laboratory findings and clinical progression, including severe bleeding and failure of FV recovery despite replacement, ultimately led to the suspicion of a non‐neutralizing FV inhibitor.

This evolving picture prompted the initiation of immunosuppressive therapy. Rituximab (375 mg/m^2^) was given on Day 9 to target B‐cell autoantibody production, along with 1 gm/kg IVIG on Days 13 and 15. This led to FV levels improving to 15% and INR stabilizing at 1.5. Anemia was treated with a total of 9U PRBC required during hospitalization with improvement and stability in hemoglobin; transfusions were not required after IVIG was administered. Hematuria resolved, and coagulation parameters improved, allowing bladder irrigation to be discontinued by Day 12.

The patient was discharged after completing the initial rituximab course, with INR at 1.5, hemoglobin at 10.0 g/dL, and improving FV activity. Rituximab continued as outpatient therapy, with four weekly doses followed by monthly maintenance. Follow‐up showed sustained improvement in FV levels (76%) and full symptom resolution.

## 3. Discussion

FV is essential for the prothrombinase complex, accelerating prothrombin to thrombin conversion by Factor Xa, in the presence of calcium and phospholipids [4]. FV deficiency is a rare bleeding disorder, which may be genetic or acquired. When inherited genetically, it is called parahemophilia or Owren’s disease [5]. Inherited mutations in the F5 gene, which encodes FV, are the primary cause [6]. Isolated or single acquired FV deficiency can also be acquired, most commonly due to inhibitors against FV.

Inhibitors to FV can develop in various conditions: Spontaneous autoantibodies may form after exposure to bovine thrombin that has been contaminated with bovine FV, or in individuals with FV deficiency who have received plasma therapy [7]. They can also develop due to medications, malignancies, autoimmune disorders, pregnancy, infections, or surgeries, particularly cardiovascular and abdominal, without bovine protein exposure [8, 9]. FV antibodies are usually of the IgG isotype, though IgM antibodies can also occur [1].

FV inhibitors often cause bleeding, mainly from mucosal surfaces such as the gastrointestinal and genitourinary systems. While cerebral or retroperitoneal hemorrhages may occur, hemarthroses are rare. Postoperative bleeding typically happens 7–10 days after surgery. Up to 20% of patients may be asymptomatic and diagnosed through abnormal lab results [1, 10].

Patients with FV inhibitors show prolonged aPTT and prothrombin time, with reduced or undetectable FV activity. Mixing studies reveal a gradual decline in FV activity, and inhibitor concentration is measured using the Bethesda assay. A mixing study helps differentiate between a coagulation factor deficiency and an inhibitor. Immediate and sustained correction with normal plasma indicates a deficiency, while delayed or no correction suggests an inhibitor. This distinction is important as inhibitors often neutralize clotting factors over time with incubation [11].

The severity of FV inhibitor activity does not always correlate with bleeding. Some patients with undetectable FV levels show no symptoms, while others with moderate suppression experience bleeding. This variability may be due to FV being stored in platelet alpha granules, which can protect it from inhibitors in the bloodstream [1]. A retrospective review at the Mayo Clinic found that while some cases showed immediate inhibition in mixing studies, progressive inhibition, where clotting times worsen after incubation, was not consistently observed. This suggests that some inhibitors may function through mechanisms other than direct factor neutralization [10].

In our patient, plasma therapy showed no response. This is likely because the FFP contained insufficient FV to overcome inhibition by rapid clearance. Platelet transfusion resolved bleeding, given the presence of activated intraplatelet FV [12]; however, this effect was temporary, as the FV was cleared by the inhibitor coagulation abnormalities persisted. While a lack of FV has been demonstrated in stored platelets [13], FFP did not resolve the patient’s bleeding either—implicating inhibition. Mixing studies initially suggested a deficiency, as they showed correction ex vivo. However, the lack of response to factor replacement raised suspicion for a non‐neutralizing FV inhibitor. Based on this, the patient was treated with IVIG and immunosuppression, which ultimately resolved the symptoms as well as the coagulation lab parameters. A previous case of detectable FV inhibition was found to be refractory to Factor Eight Inhibitor Bypassing Activity (FEIBA) treatment [14]. It is likely that FEIBA administration in our described case would have yielded a similar result: Mechanistically, FEIBA requires adequate downstream functional FV to support thrombin generation.

We hypothesize a non‐neutralizing FV inhibitor, which accelerates its clearance from the body without impairing its function, leading to a deficiency. While this mechanism is not widely reported in acquired FV inhibition, similar clearance‐based phenomena have been documented in other rare coagulopathies. For instance, non‐neutralizing antibodies to prothrombin have been shown to accelerate the plasma clearance of prothrombin without impairing its function, causing hypoprothrombinemia and bleeding [15]. Similarly, Factor X deficiency in systemic amyloidosis is attributed to sequestration and rapid clearance rather than direct inhibition [16]. Type 2 or non‐neutralizing inhibitors have been reported with other factors such as Factor VIII as well. These inhibitors are antibodies that partially neutralize or do not fully block clotting factor activity [17]. Non‐neutralizing inhibitors bind to specific epitopes on clotting factors, changing their shape and making them more detectable by the immune system. This increases clearance through the liver and spleen, shortening the clotting factor’s half‐life. As a result, patients may require higher or more frequent doses of clotting factor replacement, complicating hemophilia management [18–20].

Although FV antigen levels were not measured, the persistently undetectable FV activity despite plasma infusion, combined with clinical bleeding and response to immunosuppression, supports the hypothesis of a non‐neutralizing inhibitor causing accelerated clearance. The absence of antigen testing is a limitation in definitively confirming this mechanism, but the constellation of findings remains highly suggestive of this pathophysiology.

The management of FV inhibitors aims to control bleeding and eliminate the autoantibody. Asymptomatic individuals do not need treatment, while bleeding patients may receive FFP or platelet transfusions. A review found that 71% of patients responded well to platelet transfusions [21]. In our case, platelet transfusions provided only temporary bleeding control. Immunosuppressive regimens, such as corticosteroids alone or in combination with cyclophosphamide, were reported to suppress autoantibodies [22, 23]. However, corticosteroids were avoided in our patient due to his advanced age and the risk of side effects. Instead, rituximab and IVIG, which have been reported to be effective in FV inhibitors [24–26], led to sustained improvement in FV levels and resolution of bleeding in our patient. rFVIIa, as a bypassing agent, has been successfully used in several cases [27, 28], though in our case, rFVIIa had limited success, necessitating a shift in treatment strategy. Plasmapheresis has also been shown to reduce antibody levels, improving the effectiveness of antihemorrhagic and immunosuppressive treatments [21, 29], though it was not utilized in our patient’s management. Finally, recently approved coagulation rebalancing agents for treatment of Hemophilia A and B may prove helpful in some rare bleeding disorders for which there are no available factor concentrates, such as inherited or acquired FV deficiency. For a case such as ours, the antithrombin siRNA fitusiran would be the most likely to be of benefit. The anti‐TFPI agents concizumab or Marstacimab, which work by reducing FX inhibition by TFPI, are unlikely to work in severe FV deficiency of any cause, since both FX and FV are required for activation of prothrombin to thrombin [30]. An additional reason anti‐TFPI agents may not work in our case is that FV may function as a carrier for TFPI [31]; any reduction in FV levels results in a corresponding decrease in TFPI levels [32]. Regardless, use of these agents outside of hemophilia would be off label and is best done in the context of a clinical trial, especially given the potential thrombotic risk associated with their use.

## 4. Conclusion

This case may be one of the first descriptions of a potential non‐neutralizing, clearance‐driven FV inhibitor that corrects in mixing studies, emphasizing the importance of clinical context in interpreting coagulation tests.

It highlights a diagnostic challenge in acquired coagulopathies, as non‐neutralizing inhibitors can mimic factor deficiencies, especially when mixing studies show complete correction. Our patient had undetectable FV activity but a normal mixing study, initially suggesting a deficiency. This could have led to excluding an inhibitor from the diagnosis, especially in nonspecialized settings. In cases mimicking factor deficiency where coagulopathy does not improve with factor replacement, physicians should consider non‐neutralizing inhibitors as a diagnosis of exclusion. Prompt immunosuppression may correct observed coagulopathies and prevent life‐threatening complications.

## Disclosure

All authors approved the final version.

## Conflicts of Interest

The authors declare no conflicts of interest.

## Author Contributions

Shreyas Kalantri and Pranali Pachika drafted the manuscript. Shiva Balasubramanian assisted with editing and revising the manuscript as well as refining figures. Bayan Alquran created the figures. Vivek Sharma and Morgan McCoy provided senior supervision, critical review, and substantive edits. All authors contributed to the revision of the manuscript.

## Funding

The author received no specific funding for this work.

## Supporting Information

Supporting Table 1 summarizes the patient’s treatment course, including the timing of therapeutic interventions and corresponding changes in coagulation parameters. This table is provided as supporting information to complement Figure 1 and to avoid redundancy in the main manuscript.

## Supporting information


**Supporting Information** Additional supporting information can be found online in the Supporting Information section.

## Data Availability

The data that support the findings of this study are available from the corresponding author upon reasonable request.
