# Supplementary material for: A Case Report of Non‐Neutralizing Acquired Factor V Inhibitor Mimicking Deficiency: Diagnostic Challenges and Therapeutic Implications
Source: Case Rep Hematol. 2026 Jan 8;2026:4306324. doi: 10.1155/crh/4306324 (PMC12783055; doi:10.1155/crh/4306324)
Supplement: Supplementary file 1 — Supporting Information Additional supporting information can be found online in the Supporting Information section. [file CRH-2026-4306324-s001.pptx]

## Slide 1
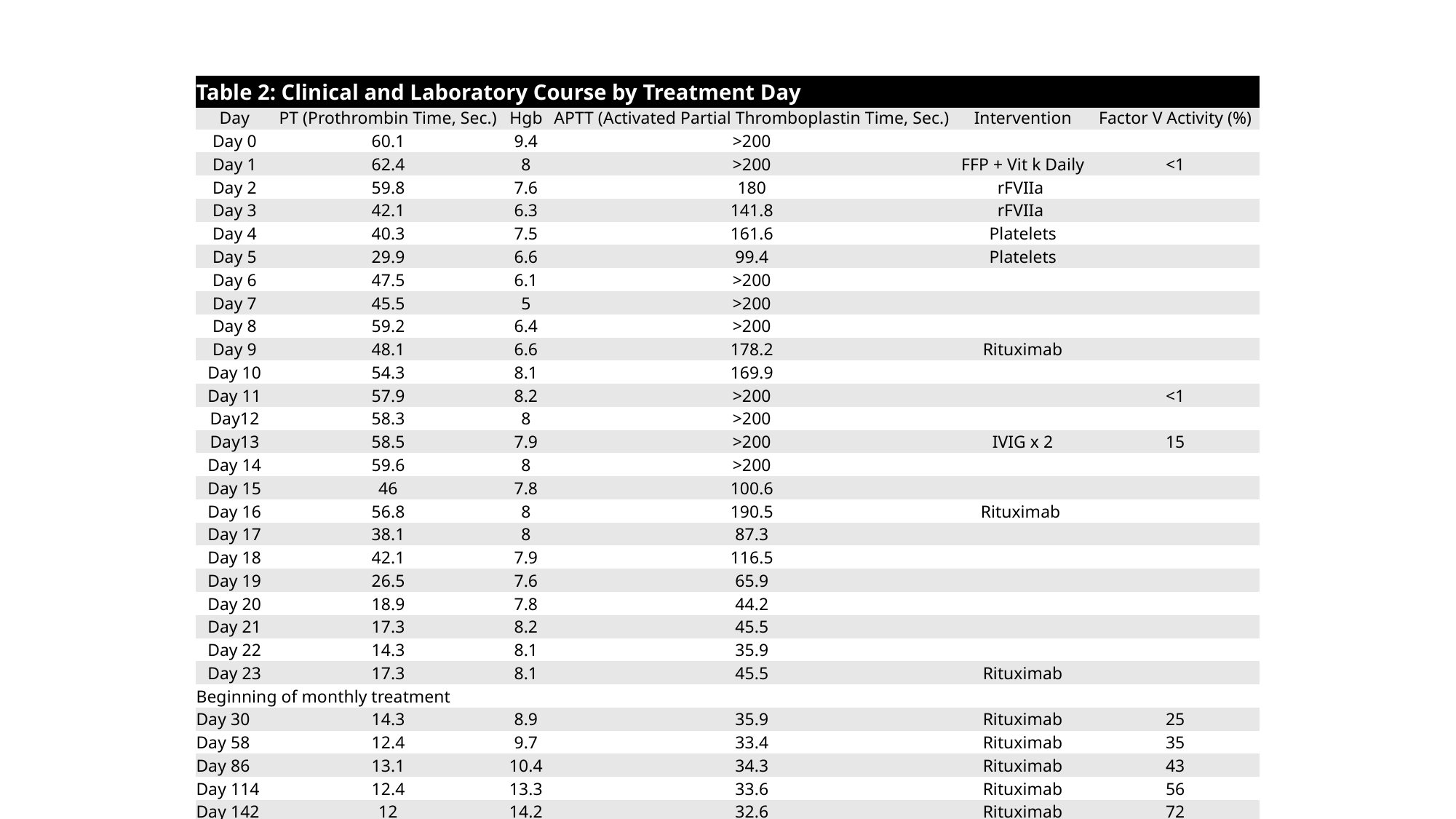

| Table 2: Clinical and Laboratory Course by Treatment Day | | | | | |
| --- | --- | --- | --- | --- | --- |
| Day | PT (Prothrombin Time, Sec.) | Hgb | APTT (Activated Partial Thromboplastin Time, Sec.) | Intervention | Factor V Activity (%) |
| Day 0 | 60.1 | 9.4 | >200 | | |
| Day 1 | 62.4 | 8 | >200 | FFP + Vit k Daily | <1 |
| Day 2 | 59.8 | 7.6 | 180 | rFVIIa | |
| Day 3 | 42.1 | 6.3 | 141.8 | rFVIIa | |
| Day 4 | 40.3 | 7.5 | 161.6 | Platelets | |
| Day 5 | 29.9 | 6.6 | 99.4 | Platelets | |
| Day 6 | 47.5 | 6.1 | >200 | | |
| Day 7 | 45.5 | 5 | >200 | | |
| Day 8 | 59.2 | 6.4 | >200 | | |
| Day 9 | 48.1 | 6.6 | 178.2 | Rituximab | |
| Day 10 | 54.3 | 8.1 | 169.9 | | |
| Day 11 | 57.9 | 8.2 | >200 | | <1 |
| Day12 | 58.3 | 8 | >200 | | |
| Day13 | 58.5 | 7.9 | >200 | IVIG x 2 | 15 |
| Day 14 | 59.6 | 8 | >200 | | |
| Day 15 | 46 | 7.8 | 100.6 | | |
| Day 16 | 56.8 | 8 | 190.5 | Rituximab | |
| Day 17 | 38.1 | 8 | 87.3 | | |
| Day 18 | 42.1 | 7.9 | 116.5 | | |
| Day 19 | 26.5 | 7.6 | 65.9 | | |
| Day 20 | 18.9 | 7.8 | 44.2 | | |
| Day 21 | 17.3 | 8.2 | 45.5 | | |
| Day 22 | 14.3 | 8.1 | 35.9 | | |
| Day 23 | 17.3 | 8.1 | 45.5 | Rituximab | |
| Beginning of monthly treatment | | | | | |
| Day 30 | 14.3 | 8.9 | 35.9 | Rituximab | 25 |
| Day 58 | 12.4 | 9.7 | 33.4 | Rituximab | 35 |
| Day 86 | 13.1 | 10.4 | 34.3 | Rituximab | 43 |
| Day 114 | 12.4 | 13.3 | 33.6 | Rituximab | 56 |
| Day 142 | 12 | 14.2 | 32.6 | Rituximab | 72 |
| Day 170 | 11.9 | 14.5 | 34 | Rituximab | 73 |
| Day 198 | 11.5 | 14.4 | 28.3 | Rituximab | 74 |
| Day 226 | 11.6 | 14.8 | 31.6 | Rituximab | 76 |
